# Acute Epiglottitis Secondary to the Severe Acute Respiratory Syndrome Coronavirus 2: A Case Report

**DOI:** 10.5811/cpcem.1581

**Published:** 2023-08-01

**Authors:** Melissa Gunchenko, Mohammad Abboud, Zachary W. Risler, Ryan C. Gibbons

**Affiliations:** * Lewis Katz School of Medicine at Temple University, Department of Emergency Medicine, Philadelphia, Pennsylvania; † Nazareth Hospital, Department of Emergency Medicine, Philadelphia, Pennsylvania

**Keywords:** airway emergencies, epiglottitis, coronavirus disease 2019, case report

## Abstract

**Introduction:**

Acute epiglottis is a rapidly progressive, potentially life-threatening infection causing inflammation of the epiglottis and adjacent supraglottic structures.^1^–^2^ Since the introduction of the *Haemophilus influenzae* vaccine, the incidence of pediatric cases has decreased dramatically while adult instances have increased.^1^–^4^ Likewise, the etiology has changed considerably with the increasing prevalence of other causative bacterial and viral pathogens.^1^–^4^

**Case Report:**

We present a novel case of acute epiglottis secondary to infection with the severe acute respiratory syndrome coronavirus 2. This case report highlights the changing landscape of epiglottitis and the importance of airway assessment.

**Conclusion:**

Present-day epiglottitis differs greatly from our traditional understanding. Numerous etiologies beyond *Haemophilus influenzae* now afflict adults predominately. As a clinically significant, novel complication of coronavirus disease 2019, acute epiglottitis is a life-threatening airway emergency. Emergency physicians must maintain a high index of suspicion, especially given the evolving clinical landscape. Early airway assessment with nasopharyngolaryngoscopic is critical.

## INTRODUCTION

Acute epiglottis is a rapidly progressive, potentially life-threatening infection causing inflammation of the epiglottis and adjacent supraglottic structures.^1^–^2^ Since the introduction of the *Haemophilus influenzae* type b (HiB) vaccine, the incidence of pediatric cases has decreased dramatically while adult instances have increased.^1^–^4^ Likewise, the etiology has changed considerably with the increasing prevalence of other causative bacterial and viral pathogens.^1^–^4^

The novel viral illness coronavirus 2019 (COVID-19), caused by the severe acute respiratory syndrome coronavirus 2, has become a global pandemic infecting over 676 million people worldwide since December 2019.^5^ While extensive pulmonary disease is a known sequela of COVID-19 infection, extrapulmonary manifestations have been observed as well, including coagulopathies and cardiovascular complications.^6^–^8^ This is only the second case report in the emergency medicine (EM) literature of COVID-19 epiglottitis, a novel complication with particular relevance to EM practice.^9^–^12^ Furthermore, it highlights the importance of emergency physician airway assessment and management.

## CASE REPORT

A 25-year-old male presented to an outside emergency department (ED) with gradual onset flu-like symptoms, odynophagia, and shortness of breath over the preceding three to four days. Initial physical examination revealed no uvular swelling, oropharyngeal exudate, stridor, wheezing, or respiratory distress. Inflammatory markers were notable for a leukocytosis of 21.5 thousand per cubic millimeter (K/mm^3^) (reference range 4–11 K/mm^3^) and an elevated C-reactive protein of 11.8 milligrams per deciliter (mg/dL) (0.0–0.4mg/dL). The initial treating clinician suspected a deep space neck infection and ordered computed topography (CT) imaging of the neck, which demonstrated edematous thickening of the epiglottis and mucosal hyperemia consistent with acute epiglottitis. Given a reported penicillin allergy, the patient received intravenous (IV) clindamycin (600 mg) and was transferred to our academic institution for otolaryngology evaluation.

On arrival, the patient was tachycardic but otherwise hemodynamically stable. Examination was notable only for a muffled voice. The emergency physician (EP) performed an immediate bedside nasopharyngolaryngoscopic (NPL) examination. Given the concern for imminent airway compromise, the EP preloaded a 6-0 endotracheal tube onto the scope. The NPL demonstrated severe swelling of the epiglottis and arytenoids with near complete upper airway obstruction (Video 1). The EP performed an immediate awake intubation using ketamine (2mg/kilogram [kg] IV). Following intubation, the patient received dexamethasone (10 mg IV), vancomycin (20 mg/kg IV), and ceftriaxone (2 grams IV) and was admitted to the intensive care unit. Prior to administering ceftriaxone, the EP confirmed a remote history of a questionable rash to penicillin. Therefore, the EP deemed the cephalosporin safe and appropriate.

The following lab tests were negative: HIV types 1 and 2 antibody and p24 antigen; hepatitis B viral surface antigen; hepatitis C viral antibody and ribonucleic acid; streptococcus rapid screen; monoscreen; blood, throat, and sputum cultures; and rapid influenza type A and B antigens. A respiratory pathogen panel was negative for other viruses. A nasopharyngeal reverse-transcription polymerase chain reaction swab (Luminex Corporation, Austin, TX) was positive for COVID-19.

The patient improved clinically and was extubated on hospital day five. The inpatient team discontinued the steroid and antibiotics and discharged the patient home on hospital day seven without complications.

## DISCUSSION

Prior to the advent of the HiB vaccination, traditional doctrine considered epiglottitis a predominately pediatric diagnosis. However, acute epiglottitis is now two to eight times more common in adults.^2^ Currently *Streptococcus pneumoniae* and *Staphylococcus aureus* are the most frequent causative agents, typically secondary to direct inoculation or bacteremia.^1^–^3^,^14^,^15^ Other less common etiologies include fungal and viral organisms, granulomatous and lymphoproliferative conditions, traumatic and thermal injuries, and toxic ingestions.^1^–^3^,^14^

Adult epiglottitis presents a diagnostic challenge. Given the anatomic airway dissimilarities, children typically present in respiratory distress with drooling and in a tripod position, whereas adults demonstrate less severe atypical signs and symptoms^1^–^2^ with sore throat, odynophagia, and dysphagia being the most common.^1^–^4^,^13^–^15^ Stridor, drooling, and voice changes are present in fewer than 60% of cases.^1^–^4^,^13^–^15^ Furthermore, adults have a more indolent course compared to the acute decompensation common among the pediatric population.^2^,^14^ Our patient presented similarly. Physical examination and plain films lack adequate sensitivity.^1^–^3^,^12^ In fact, lateral neck films have a false negative rate over 30%, and the classic “thumbprint sign” is present in less than 80% of cases.^1^,^3^,^14^,^16^ While computed tomography with intravenous contrast of the neck has sensitivities between 88–100%, patients may be too unstable for transport.^3^,^13^,^16^,^17^ Nasopharyngeal laryngoscopy has a sensitivity of 100% and facilitates assessment of disease severity as well.^3^,^13^,^14^,^18^

**CPC-EM Capsule**

What do we already know about this clinical entity?

*Airway compromise is a known complication of epiglottitis and is associated with a higher morbidity and mortality.*

What makes this presentation of disease reportable?

*Epiglottitis secondary to coronavirus disease 2019 is an emerging complication of which emergency physicians (EPs) need to be aware.*

What is the major learning point?

*Since the advent of the* Haemophilus influenzae *type b vaccine, the clinical presentation of epiglottitis has changed. Adults are more frequently affected and often have atypical presentations.*

How might this improve emergency medicine practice?

*Increased awareness of the changing clinical landscape of epiglottitis will facilitate EPs recognition of this clinical entity.*

Using the National Emergency Departments Sample database, Hanna et al retrospectively reviewed over 33,000 cases of adult epiglottitis from 2007–2014 and found that less than 1% of patients had a laryngoscopic assessment or airway intervention in the ED, highlighting the lack of recognition of this condition.^19^ Nonetheless, practice patterns vary. A more recent, single-center retrospective study determined that 55% of patients had an airway assessment in the ED.^13^ However, only three cases had an EP perform the evaluation.^13^ Nasopharyngolaryngoscopy is often unavailable, or training is limited in the ED. In such instances, EPs must involve otolaryngology or anesthesiology early in the assessment of suspected epiglottitis. Study results have varied, with findings that indicate 8–50% of patients will require intubation with 10–33% requiring surgical intervention.^3^,^4^,^14^,^15^,^18^ In the aforementioned single-center study, 17% of patients needed an advanced airway, 25% of which were surgical.^13^

Anticipating an advanced airway is challenging. Numerous studies have attempted to determine risk factors associated with airway interventions. Immunocompromised patients and those with diabetes, as well as patients presenting with hypoxia, stridor, voice changes, and airway edema, typically require emergent airway management.^3^,^14^,^15^,^18^,^20^ Mortality ranges between 1–20%, but upper airway obstruction is associated with a five-fold increase in mortality.^1^,^3^,^4^,^13^–^15^,^19^–^20^ Therefore, anticipating an airway emergency is critical in suspected cases of epiglottitis.

## CONCLUSION

Present-day epiglottitis differs greatly from our traditional understanding. Numerous etiologies beyond *H. influenzae* now afflict adults predominately. As a clinically significant, novel complication of COVID-19, acute epiglottitis is a life-threatening airway emergency. Emergency physicians must maintain a high index of suspicion, especially with the changing clinical landscape. Early airway assessment is critical in suspected cases.

## Supplementary Information

Video 1 Nasopharyngolaryngoscopic video of edematous epiglottis and arytenoids obscuring visualization of airway.
